# Autism-associated synaptic vesicle transcripts are differentially expressed in maternal plasma exosomes of physiopathologic pregnancies

**DOI:** 10.1186/s12967-021-02821-6

**Published:** 2021-04-15

**Authors:** Yangwu Fang, Chan Wan, Youlu Wen, Ze Wu, Jing Pan, Mei Zhong, Nanbert Zhong

**Affiliations:** 1 Department of Obstetrics and Gynecology, Nanfang Hospital, Southern Medical University, 1838 North Guangzhou Avenue, Guangzhou, 510515 China; 2 Sino-US Center of Translational Medicine for Development Disabilities, Southern Medical University, Guangzhou, 510515 China; 3 Department of Psychiatry, Guangdong 999 Brain Hospital, Guangzhou, 510510 China; 4 Department of Human Genetics, New York State Institute for Basic Research in Developmental Disabilities, 1050 Forest Hill Rd, Staten Island, NY 10314 USA

**Keywords:** Synapse, Spontaneous preterm birth, Preeclampsia, Gestational diabetes mellitus, Exosomes

## Abstract

**Background:**

During intrauterine development, the formation and function of synaptic vesicles (SVs) are thought to be fundamental conditions essential for normal development of the brain. Lacking advanced technology during the intrauterine period, such as longitudinal real-time monitoring of the SV-associated transcripts (SVATs), which include six pairs of lncRNA-mRNA, has limited acquisition of the dynamic gene expression profile (GEP) of SVATs. We previously reported the differential expression of SVATs in the peripheral blood of autistic children. The current study was designed to determine the dynamic profiles of differentially-expressed SVATs in circulating exosomes (EXs) derived from autistic children and pregnant women at different gestational ages.

**Methods:**

Blood samples were collected from autistic children and women with variant physiopathologic pregnancies. EXs were isolated with an ExoQuick Exosome Precipitation Kit and characterized by transmission electron microscopy (TEM), nanoparticle tracking analysis (NTA), and Western blotting. The expression of lncRNAs and lncRNA-targeted mRNAs were quantified using real-time PCR.

**Results:**

SVAT-associated lncRNAs-mRNAs were detected in autistic children and differentially expressed from the first trimester of pregnancy to the term of delivery. Pathologic pregnancies, including spontaneous preterm birth (sPTB), preeclampsia (PE), and gestational diabetes mellitus (GDM), were compared to normal physiologic pregnancies, and shown to exhibit specific correlations between SVAT-lncRNA and SVAT-mRNA of *STX8*, *SLC18A2*, and *SYP* with sPTB; SVAT-lncRNA and SVAT-mRNA of *STX8* with PE; and SVAT-lncRNA and SVAT-mRNA of *SV2C* as well as SVAT-mRNA of *SYP* with GDM.

**Conclusion:**

Variant complications in pathologic pregnancies may alter the GEP of SVATs, which is likely to affect the intrauterine development of neural circuits and consequently influence fetal brain development.

**Supplementary Information:**

The online version contains supplementary material available at 10.1186/s12967-021-02821-6.

## Background

Synapses, which are composed of presynaptic and posterior synapses, are specialized junctions between neurons in the brain that relay and process information. Synapses connect neurons into the intricate neural network of neural circuits, which are the fundamental elements that induce brain function [1]. The concept of neural circuits serves as a helpful tool to probe into the mechanism underlying brain function; however, it is crucial to take into consideration the limitations of the concept when assessing the function of synapses in neurologic processing. The coexistence of electrical and chemical synapses in the brain structures of most animals has been long-established, but investigation into the details of these two transmission modalities and the distribution are ongoing [2]. Chemical transmission involves complex presynaptic molecular machinery that controls the release of neurotransmitters in an orderly manner when an action potential is fired at the synaptic terminal. Thus far, exploration of the mechanisms underlying chemical transmission forms the central focus of most research [3]. The process of synaptic pruning is at the heart of our understanding of circuit constructions. Synaptic vesicles (SVs) are small carriers that are electron-lucent at presynaptic terminals. Neurotransmitters accumulate in SVs, awaiting release through Ca^2+^-triggered exocytosis [4]. SVs contain synaptic vesicle–associated transcripts (SVATs) and synaptic vesicle-associated proteins (SVAPs), which have a variety of functions, including the following functions: ion channels; scaffolding proteins; neurotransmitter receptors; and facilitating synaptic cell adhesion. SVs are produced locally at synaptic terminals and are regenerated through SV cycling (SVC) that contains proteins with two functions (the uptake of neurotransmitters and membrane trafficking [5]), thus supporting both synaptic functions and structural integrity. Few studies, however, have investigated the mechanisms that induce synaptic neurotransmission and the relationship with many neurologic diseases [6].

Extracellular vesicles (EVs) are nano particles that consist of a lipid bilayer membrane and RNA, DNA, and proteins. EVs can be separated from any biological fluid and can be used to reflect the balance between uptake and secretion by different types of local cells. EVs are comprised of a variety of biomolecules, many of which are potential biomarkers for disease [7]. Exosomes (EXs), defined as small spherical EVs that are 30–150 nm in size, are a class of EVs derived from the endosome/multivesicular body system. EXs are responsible for selectively transferring biologically-active materials between cells, i.e., EXs are involved in intercellular communications. Recent findings have demonstrated the transport and transfer of proteins and miRNA via EXs, indicating that EXs are potential effectors in diseases and innovative biomarkers for diagnosing, predicting prognoses, and treating diseases [8].

RNA participates in numerous biological activities. Many types of RNA exist within the human body, among which messenger RNA (mRNA, with an average length of 1000–1500 nucleotides), microRNA (miRNA, with an average length of ~ 20 nucleotides), and long non-coding RNA (lncRNA, with an average length ≥ 200 nucleotides) are the most widely investigated as a result of the wide distribution [9]. Recently, lncRNAs have been shown to be present in genomes as intronic, antisense, or large intergenic non-coding RNAs (ncRNAs). In addition, lncRNAs are located at regions associated with promoters or untranslated, functioning as *cis-* or *trans-*regulators. LncRNAs may serve as a transcriptional and post-transcriptional regulators of neural development and cell differentiation, hence lncRNAs are related to many human neural disorders [10], including psychiatric and neurodegenerative disorders, immune dysfunction, autoimmune diseases, cardiovascular disease, cancer, and reproductive disorders [11]. LncRNA deregulation is gaining recognition because many pathologic processes bear this characteristic. Moreover, lncRNAs associated with malignant tumors may be utilized as diagnostic or prognostic biomarkers, and potentially as targets of selective silencing of innovative therapeutic agents [12].

EXs are produced by every cell type within the central nervous system, these including basic neurons, oligodendrocytes, microglia, astrocytes [13], and neural stem cells. Neuron-derived EXs can be detected in peripheral blood plasma [14]. Fetal EXs can also enter the maternal circulatory system via the placenta [15]. Therefore, detecting different components of fetal EXs in the maternal blood circulation can provide new indicators of prenatal fetal development, including fetal brain development. In our previous sequencing studies on autism in children, altered expression of lncRNAs and mRNAs was documented to be associated with SV trafficking [16]. The present study aimed to investigate the differentially-expressed patterns of lncRNA-mRNA SVATs in plasma EXs from children and maternal peripheral blood at different gestational ages. The differences in expression of these molecules between EXs of healthy pregnant women and women with pathologic pregnancies, including spontaneous preterm birth (sPTB) resulting from spontaneous preterm labor (sPTL) or preterm premature rupture of membrane (pPROM), preeclampsia (PE), or gestational diabetes mellitus (GDM), were assessed to identify molecular candidates as early predictive biomarkers.

## Methods

### Sample collection

This study was approved by the Medical Ethics Committee of Guangdong Triple-Nine Brain Hospital (recruitment of 14 children with autism spectrum disorder [ASD] and 14 healthy children, 2–10 years of age) and Nanfang Hospital of Southern Medical University (recruitment of pregnant women). Informed consent was obtained from the pregnant women and the guardians of the children. The peripheral blood of pregnant women was collected, as follows: 24^+0^–32^+6^ gestational weeks (GWs [n = 20]); and 33^+0^–39^+6^ GWs (n = 20). EXs were isolated from plasma samples and subjected to RNA-seq. A minimum of 30 healthy pregnancies collected on a weekly basis at each GW from the 11^th^- 40^th^ GW were subjected to SVATs assessment. Plasma from 38 sPTBs, 34 women with GDM, and 19 women with PE in the third trimester (34 GW for sPTB and 34–35 GW for PE and GDM) were retrospectively used as case-controls.

### EX isolation

Peripheral blood was collected into EDTA tubes (BD, New York City, NY, USA). After the blood was collected, the tubes were inverted five times, stored on ice, and processed within 30 min. Each specimen was centrifuged at 1500*g* for 15 min at 4 °C to separate plasma from cells. The plasma was stored at − 80 °C until processing of EX isolation, for which 550 μL of plasma was incubated with 5.5 μL of thrombin (TMEXO-1; System Biosciences, Mountain View, CA, USA) at room temperature for 5 min, then centrifuged in a microcentrifuge at 10,000*g* for 5 min. Approximately 500 μL of supernatant was transferred into a sterile vessel and incubated with 120 μL of ExoQuick Exosome Precipitation Solution (EXOQ5TM; System Biosciences) and 5.5 μL-cocktails of protease and phosphatase inhibitors (PM6020; Huabodeyi, Beijing, China) at 4 °C for 30 min, followed by centrifugation at 300*g* for 30 min at 4 °C. EX pellets were resuspended in 100 μL of PBS (Gibco, Grand Island, NY, USA) and stored at − 80 °C until RNA was isolated. The concentration of total EXs was determined using a BCA Protein Assay (Thermo Fisher, Waltham, MA, USA).

### Transmission electron microscopy

Pellets of freshly-isolated EXs suspended in PBS buffer were dropped onto a carbon-coated copper grid, dried, then stained with 1% uranyl acetate (Sigma-Aldrich, St. Louis, MO, USA) for 5 min. The grids were imaged under a Hitachi H-7650 transmission electron microscope (Hitachi, Tokyo, Japan).

### Nanoparticle tracking analysis (NTA)

An aliquot of the EX suspension (10 μL of vesicles in 90 μL of buffer) was collected after adding 10 μL of 10% glycine, which was then diluted with PBS to a final ratio of 1:200 before lysis with Mammalian Protein Extraction Reagent (Millipore, Billerica, MA, USA). The mean concentration (particles/mL) and diameter (nm) of EXs were calculated using NTA 3.1 nanoparticle tracking software and the Nanosight NS300 system with a G532-nm laser module (Malvern Instruments, Malvern, UK).

### Western blotting

To confirm the success of EX isolation, ~ 30–40 μg of exosomal protein was loaded onto a polyacrylamide gel (5% stacking and 10% resolving gel). After running the gel at 90 V for 20 min, followed by 120 V for 60 min in running buffer, the proteins were transferred to a polyvinyl fluoride membrane (0.45 μm; Millipore) and blocked with 5% skim milk (BD, Lincoln, NE, USA) for 2 h. The membranes were incubated overnight with primary antibodies (Abs) against CD63, CD9, C81 (rabbit pAb; SBI, Palo Alto, CA, USA), pregnancy-specific globin (PSG1), nestin (NES), progesterone receptor [PGR] (rabbit pAb; Absin, Shanghai, China), L1 cell adhesion molecule [L1CAM] (rabbit pAb; Abcam, Cambridge, UK), and placental alkaline phosphatase [PLAP](mouse mAb; Santa Cruz Biotechnology, Inc., Santa Cruz, CA, USA) at 4 °C. The Ab dilution ranged from 500- to 1000-fold times, as appropriate. The next day, the membranes were washed and incubated with secondary antibodies (goat anti-rabbit-HRP; Beyotime, Shanghai, China). The blotted proteins were visualized using an enhanced chemiluminescence kit (Pierce Biotechnology, Rockford, IL, USA), and the signal was detected using Saga (Sage Sciences, Lincoln, NE, USA).

### RNA extraction and quantitative real-time PCR

Total RNA extraction from EXs was achieved using TRIzol (Invitrogen, Carlsbad, CA, USA). The HiScript Reverse Transcription Kit and ChamQ SYBR Green qPCR Kit (Vazyme, Nanjing, China) were used to perform reverse transcription and quantify RNAs in accordance with the manufacturer’s instructions. The levels of lncRNA-targeted mRNA and lncRNA expression were established using quantitative real-time PCR (the primer sequences used in quantitative PCR are listed in Additional file 1: Tables S1) using a LightCycle 96 system (Roche, Grand Island, NY, USA). U6 RNA was utilized as a control for the numerical analysis. The quantity of mRNA and lncRNA was normalized to the U6 level. Triplet quantitative measurement for each lncRNA or mRNA was performed. A relative normalized expression of each sample was determined based on the level of sample expression at 11 GW.

### Data analysis

All triplet analysis data are presented as the mean ± SE For the relative lncRNA and mRNA quantification, the means of the control groups were set to 1, which was not displayed in the drawing. The data were subjected to one-way analysis of variance, followed by an unpaired, two-tailed t-test. Differences were considered significant at a *P* < 0.05. The figures were drawn with GraphPad Prism (version 8.0).

## Results

### Expression of exosomal SVATs in ASD

**Fig. 1:**
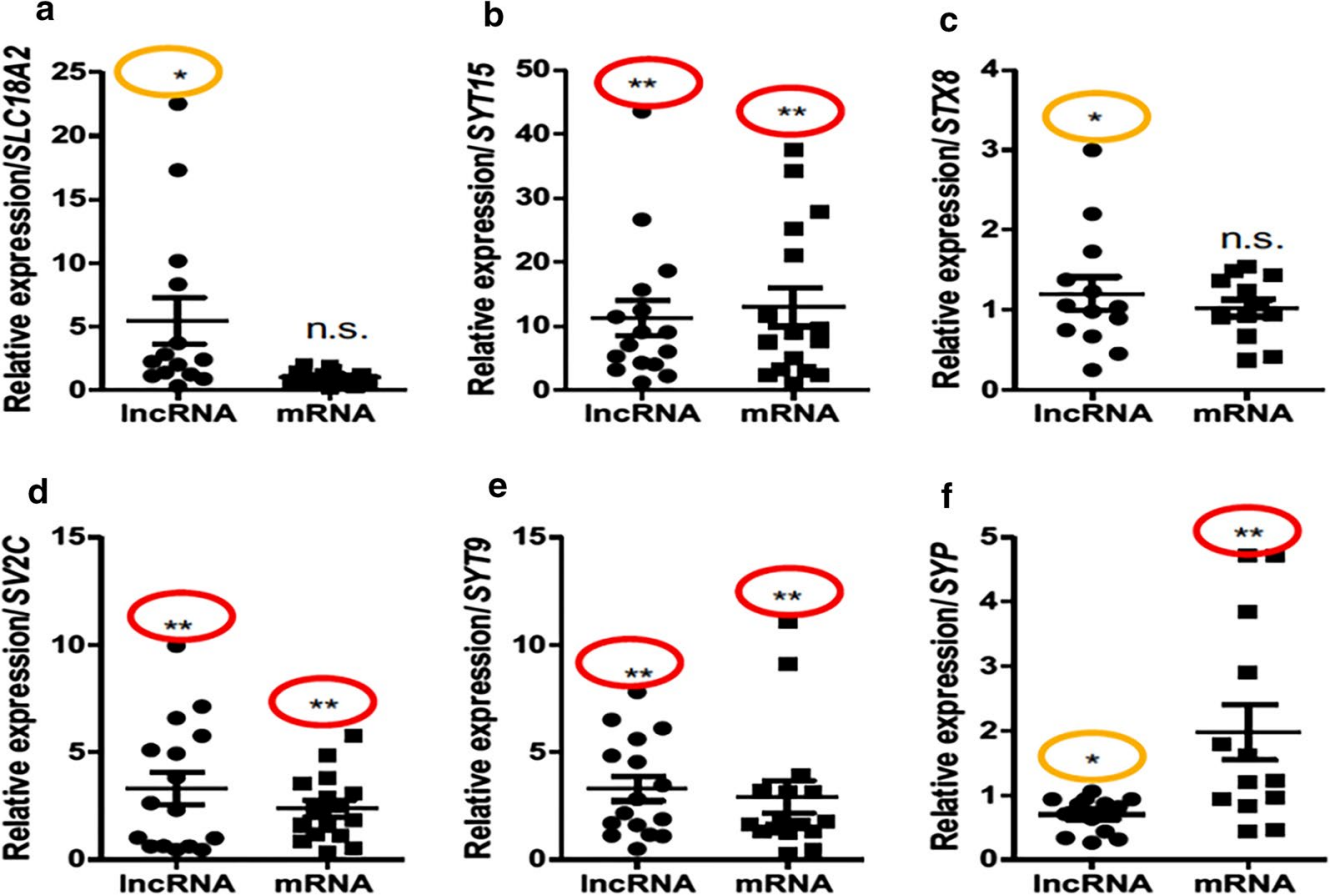
GEP of exosomal SVATs. Expression of SVATs in circulated exosomes derived from ASD children; compared to healthy children. *P < 0.05 in orange oval, **P < 0.01 in red oval

Previously, we determined the differential expression of SVATs in peripheral blood lymphocytes of children with autism [16]. In the current study, we have successfully replicated the differential expression of six SVAT pairs in peripheral lymphocytes and in circulating EXs isolated from peripheral plasma. As shown in Table 1, SVAT-lncRNAs of *SLC18A2*, *SYT9*, *STX8*, and *SYT15* were measured to be up-regulated, while SVAT-lncRNAs of *SV2C* and *SYP* were minimally expressed compared to healthy children. Differential expression profiles (DEPs) were documented in 6 SVAT-lncRNA loci and *SLC18A2*, *SYT9*, *STX8*, *SV2C*, *SYP,* and *SYT15* SVAT-mRNA (Fig. 1) when exosomal RNAs of 14 children with ASD were examined. As presented in Fig. 1, three up-regulated SVAT-mRNAs at the *SYT15* (Fig. 1b), *SYT9* (1E), and *SYP* loci (1F) and one down-regulated SVAT-mRNA at the *SV2C* (1D) locus were identified. We also noticed that 10 of 12 SVAT-associated mRNAs could not be detected from the autistic blood specimens. This finding provided evidence that RNA is very stable in EXs application which may give a better approach for testing biomarkers.

**Table 1:** GEP of SVAT-lncRNAs and SVAT-mRNAs in autism exosomes

LncRNA					mRNA				
Symbol	Blood	DEP	Exosomes	DEP	Symbol	Blood	DEP	Exosomes	DEP
**RP11-501J20.5**	Yes	Up	Yes	Up	**SLC18A2**	ND	NA	Yes	NS
**RP11-38L15.3**	Yes	Up	Yes	Up	**SYT15**	Yes	Up	Yes	Up
**STX8**	Yes	Up	Yes	Up	**STX8**	ND	NA	Yes	NS
**CTD-2516F10.2**	Yes	Up	Yes	Up	**SYT9**	ND	NA	Yes	Up
**RP11-466P24.2**	Yes	Down	Yes	Up	**SV2C**	ND	NA	Yes	Up
**SYP-AS1**	Yes	Down	Yes	Down	**SYP**	ND	NA	Yes	Up
**STX16-NPEPL1**	Yes	Up	ND	NA	**STX16**	ND	NA	ND	NA
**AC005606.14**	Yes	Up	ND	NA	**SYNGR3**	ND	NA	ND	NA
**RP1-78O14.1**	Yes	Up	ND	NA	**SYT1**	ND	NA	ND	NA
**AK128569**	Yes	Up	ND	NA	**SYT9**	ND	NA	ND	NA
**STXBP5-AS1**	Yes	Down	ND	NA	**STXBP5**	ND	NA	ND	NA
**RP5-839b4.7**	Yes	Up	ND	NA	**SNAP25**	Yes	Up	ND	NA

*DEP* differential expression profile, *Up* up-regulated, *Down* down-regulated, *ND* not detectable, *NA* not available, *NS* not significant

### Identification and characterization of EXs isolated from maternal plasma

**Fig. 2:**
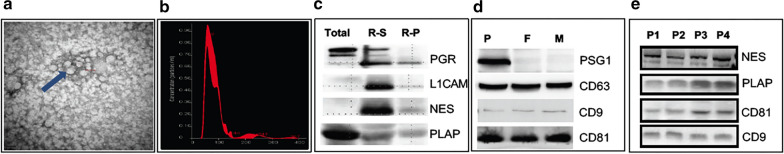
Isolation of exosomes from peripheral plasmas. Exosomes isolated from maternal plasma were observed under electronic microscopy, as pointed to by an arrow (**a**) and 50–150 nm in size determined by (**b**). Proteins loaded from total exosomes (total) and from resuspended supernatant (R-S) or resuspended pellet (R-P) when exosomes treated with lysis buffer during the process of exosomal isolation and probed with antibodies against progesterone receptor (PGR) and placental alkaline phosphatase (PLAP) (**c**, **e**). Protein extracted from the exosomes of pregnant women (P), a non-pregnant female (F), and a male (M) were probed with antibodies against pregnant specific globin 1 (PSG1) (d), CD63 (**d**), and CD9 and CD81 (**d**, **e**). The neuro-specific markers, L1CAM (L1 cell adhesion molecule) (**c**) and nestin (NES), can be detected from plasma exosomes (**c**, **e**)

EXs were isolated from plasma derived from pregnancies using a commercial kit and observed via transmission electron microscopy. The data showed that the EXs were spherical and wrapped by a double-membrane structure (Fig. 2a). The actual size of the EXs in the NTA particle size analysis system ranged from 50 to 150 nm, with most EXs observed to be < 100 nm in size (Fig. 2b). This finding was confirmed by antibodies against CD63, CD9, and CD81, which are considered to be exosomal markers (Fig. 2d, e). Antibody against PSG1 showed that EXs isolated from a pregnant woman (P) were not present in a non-pregnant female (F) and male (M), indicating that the EXs in pregnancy may be derived from pregnancy-specific tissue or cells (Fig. 2d). Indeed, PLAP, a placenta-specific marker, was shown in four EX pregnancy samples (Fig. 2e, [P1–4]). Similarly, the neuron-specific biomarkers, NES (Fig. 2c, e) and L1CAM (Fig. 2c), were also present in EXs. These results demonstrated that EXs isolated from the maternal circulation were heterogeneous and derived from placental trophoblasts as well as neurons.

### Discovery study of gene expression profiles (GEPs) of SVAT-lncRNAs and SVAT-mRNAs in pregnancies

**Fig. 3:**
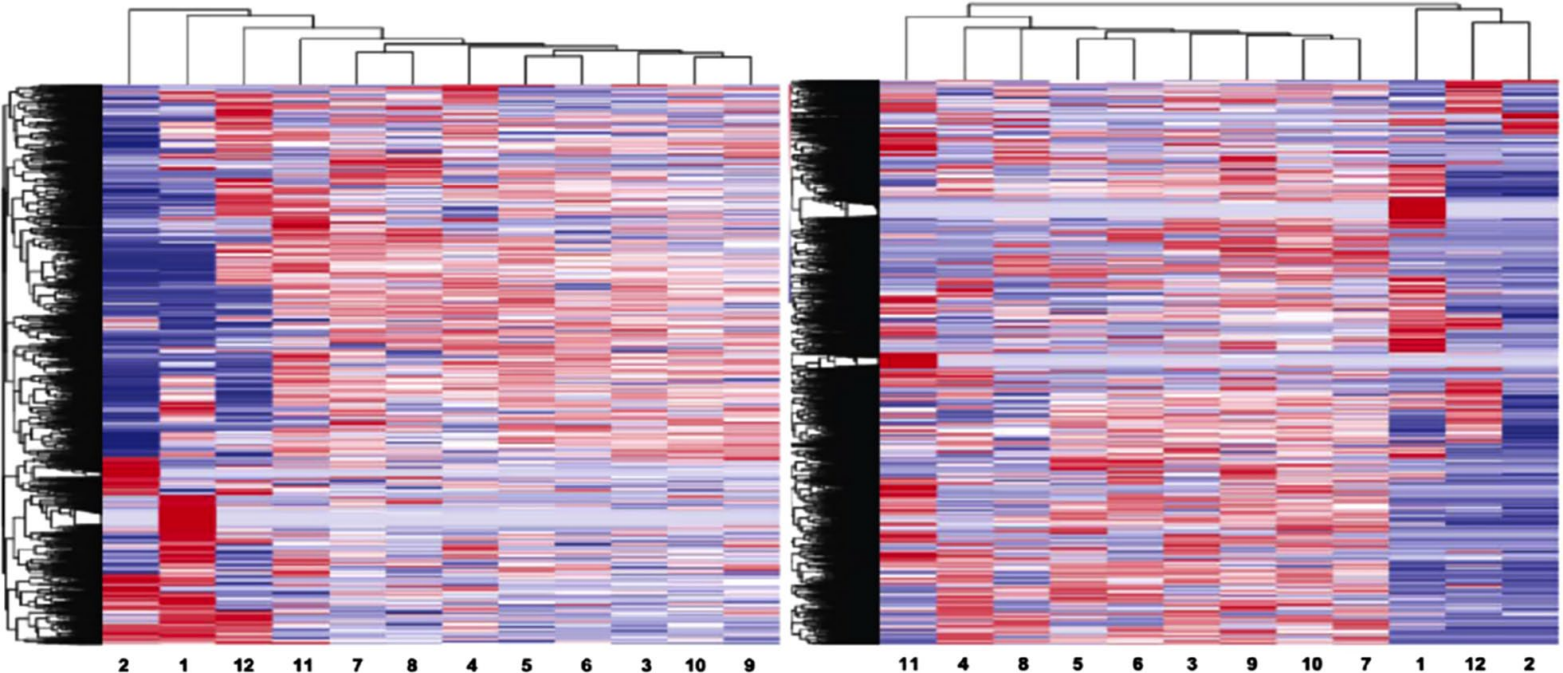
Heatmap of RN-seq profiles for exosomal lncRNAs (left panel) and exosomal mRNAs (right panel). Odd numbers represent exosome pools isolated from 5 to 8 pregnancies at 24–32 GW and even numbers represent exosome pools of 5 to 8 pregnancies at 33–39 GW, which were collected from women with spontaneous preterm birth (sPTB [lanes 1 and 2]), gestational diabetes mellitus (GDM [lanes 3 and 4]), full-term birth (FTB [lanes 5 and 6]), preeclampsia (PE [lanes 7 and 8]), preterm premature rupture of membrane (pPROM [lanes 9 and 10]), and premature rupture of membrane (PROM, [lanes 11 and 12])

To investigate GEPs of SVATs in physiopathologic pregnancies, a discovery study of SVATs, SVAT-lncRNAs and SVAT-mRNAs, was conducted by RNA-seq at two time-points of gestational ages, as presented in a heatmap (Fig. 3). Lanes 1 and 2, representing the sPTB group, have significantly different profiles compared with the other groups. The GEP at each lane represents a pool of gene expression from a mixed subset of exosomal RNAs.

### GEPs of SVATs during between the 11th and 40th GWs

**Fig. 4:**
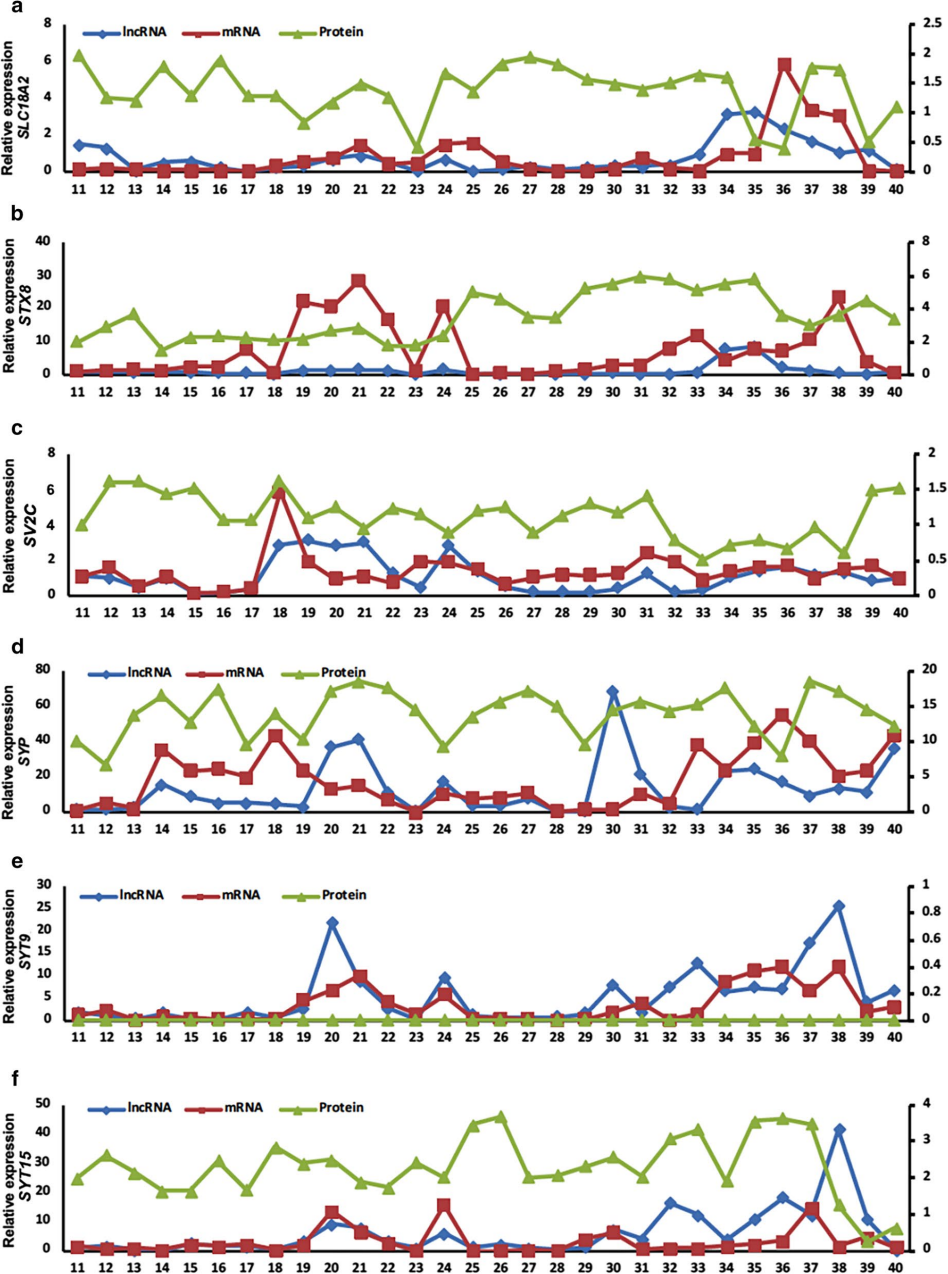
Longitudinal assessment of GEPs. Six SAVT-associated lncRNAs (blue line), mRNAs (red line), and proteins (green line) were analyzed from the 11th–40th GW during physiologic pregnancies. The GEP patterns were based on exosomal pools collected independently at every gestational week with 30 unrelated pregnancies

After demonstrating that SVATs are expressed in the 3rd trimester of physiopathologic pregnancies, we explored the GEPs of SVATs in earlier stages of pregnancy to determine the longitudinal expression from the 1^st^-3^rd^ trimesters of pregnancy. Through continuous analysis of pregnancies from the 5^th^-40^th^ GW, the GEPs of exosomal SVAT transcriptions were determined. Exosomal lncRNAs and mRNAs of all 12 pairs of SVATs were not detectable in the early stage of pregnant plasma specimens from the 5th–10th GW (data not shown). Exosomal lncRNA and mRNA of *SLC18A2* were detected from the 18th–26th GW and were highly expressed in the 3rd trimester. At the same time, lncRNA of *SLC18A2* had an apparent correlation with mRNA (Fig. 4a), unlike *STX8*. In addition, the level of *STX8* lncRNA expression was stable before the 33^rd^ GW, but the mRNA was highly expressed from the 17th–24th GW (Fig. 4b). LncRNA of *SV2C* was highly expressed in the 18th GW, whereas the mRNA had a high expression plateau from the 18th–20th GW; there was no correlation between lncRNA and mRNA of *SV2C* (Fig. 4c). The expression peak of exosomal *SYP* mRNA was from the 13th–23rd GW, followed by high expression from the 31^st^ GW. LncRNA was highly expressed between the 21st and 30th GW (Fig. 4d). Both *SYT9* lncRNA and mRNA showed an increasing trend at the 18th GW, but the level of lncRNA exhibited a decreasing trend from the peak at the 20th GW. In contrast, mRNA levels began to decrease after reaching a peak at the 21st GW, and a small peak was observed at the 24th GW. Both *SYT9* lncRNA and mRNA were highly expressed after the 33rd GW. There was a positive correlation between *SYT9* lncRNA and mRNA (Fig. 4e). The expression of *SYT15* mRNA and lncRNA in the 19th–25th GW had similar fluctuations, and these were similar in the later days of pregnancy (Fig. 4f). Except for *SYT9*, five other proteins were detected in the plasma EXs with gestational fluctuation.

### GEPs of SVAT-lncRNAs and SVAT-mRNAs in pregnancy complications

**Fig. 5:**
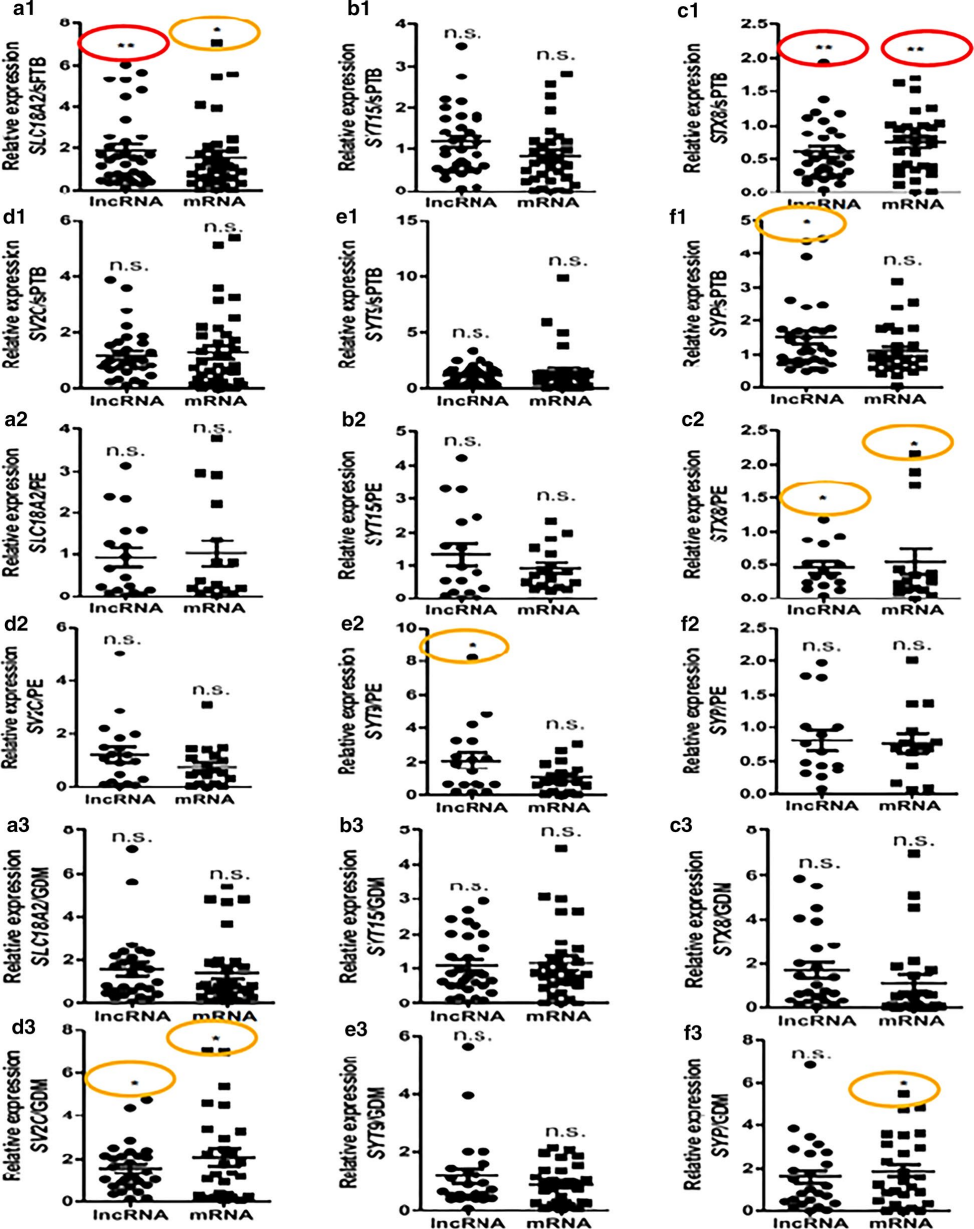
GEPs of SVAT-associated lncRNAs and mRNAs in pathologic pregnancies. sPTB: spontaneous preterm birth. PE: preeclampsia. GDM: gestational diabetes mellitus. *P < 0.05 in orange oval, **P < 0.01 in red oval

To explore the effect of pregnancy complications on lncRNA-mRNA in maternal circulating EXs, 38 cases of sPTB (28–36 GW), 19 cases of PE (28–36 GW), and 34 cases of GDM (28–39 GW) were studied. The lncRNA and mRNA expression of *SLC18A2*, *SYT15*, *STX8*, *SV2C*, *SYT9*, and *SYP* genes were detected by quantitative PCR. The results showed that *SLC18A2* lncRNA and mRNA were higher in sPTB patients than in healthy pregnant women (Fig. 5a1), while *STX8* lncRNA and mRNA were decreased (Fig. 5c1). In contrast, the expression of *SYP* lncRNA was increased (Fig. 5f1). The remaining molecules were not expressed differently between the sPTB and healthy pregnancy groups (Fig. 5b1, d1, e1, f1). In the PE group, except for decreased *STX8* lncRNA and STX8 mRNA (Fig. 5c2), there were no significant differences in gene expression for the remaining genes (Fig. 5a2, b2, d2, e2, f2). Interestingly, *SV2C* lncRNA and mRNA, as well as *SYP* mRNA, were increased in patients with GDM compared with the healthy pregnancy group (Fig. 5d3, f3). No differences in gene expression were detected between the patients with GDM and the healthy pregnancy group (Fig. 5a3, b3, c3, e3, f3).

## Discussion

This is the first study to demonstrate a connection between fetal-derived EXs and SVAT-associated lncRNAs (i.e., *SYT15*, *SYT9*, *SYP*, *SLC18A2*, *SV2C* and *STX8*; Table 1).Based on the results of this study, alteration of the lncRNA genes exists in the peripheral blood EXs of children with ASD. The expression pattern was also implied into analysis of adverse gestational outcomes, such as sPTB, PE, and GDM, although a larger size of clinical samples is required to calculate the range of lncRNA expressions in healthy newborns in relation to their health status.

SVATs are found in the CNS and peripheral circulation, playing an important role for SV release and recycling. In our previous genome-wide study of peripheral blood from children with ASD, the GEPs of lncRNAs as well as of mRNAs were identified to be differentially expressed in 12 SVATs loci [16]. The abnormal expression of SVATs often indicates abnormal development and an abnormal state of the nervous system. Abnormal expression profiles of SVATs are found in psychiatric disorders, such as autism, epilepsy, and schizophrenia [17]. Most of these disorders pose hidden risks during embryonic development. The perfection of fetal brain function during pregnancy involves a very precise process of SV cycling. EXs derived from neurons enter the circulatory system through the blood–brain barrier, including the EXs originating from fetal brain nerves. We know that EXs carry a variety of RNA, protein, and lipid molecules for long-distance signal transmission, whereas the characteristics of SVATs detected in EXs from peripheral blood remain unknown. It is a challenge to detect the functional state of neural circuits in the brain under living conditions. There is currently no effective way to respond to brain circuit function. EXs can carry Aβ proteins that are transmitted between different neurons via synapses in patients with Alzheimer’s disease [18]. The extensive activity of EXs in the nervous system suggests that EXs may be an important tool. In our study, EXs from maternal peripheral blood sources were shown to express NES and L1CAM, indicating neuronal-derived EXs. NES is primarily regarded as a marker of neural stem/progenitor cells. NES-positive cells are localized to tissue- or organ-specific sites in adults, where they serve as a quiescent resource of cells capable of proliferation, differentiation, and migration after re-activation [19]. The re-expression or upregulation of NES was observed in various tissues during repair processes after several types of injury, including the following: in reactive astrocytes after CNS damage [20]; during the fibrotic response to ischemic heart disease [21]; and in the context of mesangial cell repopulation after induced nephritis [22]. Therefore, most of the NES detected in healthy pregnant women is derived from embryonic neural precursor cells. L1CAM in neurons is essential in the neuronal development of mice, where L1CAM governs neuronal outgrowth, migration, and axonal guidance [23]. L1CAM is a transmembrane protein belonging to the immunoglobulin superfamily that interacts with several ligands, such as integrins, neuropilins, contactin 1 and 2, and CD24, as well as with the shed form [24]. In disease-free adults, L1CAM was observed exclusively in kidney epithelia [25]. Although we cannot rule out that the EXs are secreted by maternal neurons, EXs still contain fetal neurons. Through the assessment of the maternal neurologic function, the detection of abnormalities in pregnant women can also assess the synaptic function of fetal brain neurons.

Membrane-bound and secretory proteins are modified in the endosome reticulum (ER) and are essential for proper function of proteins by folding [26]. Previous studies have established that early endosomes arise from primary endocytic vesicles, which fuse with each other to form a larger endocytic structure [27]. It is likely that endocytic vesicles containing internalized EXs fuse with endocytic vesicles containing different cargoes and during endosome maturation towards the generation of intraluminal nanovesicles, which result in late endosome sheltering of endogenous vesicles and EXs taken up from another cell. It can be assumed that most of the internalized EXs are used by the cell by undergoing lysosomal degradation or recovery of some components via the trans-Golgi network [28].

The results in this study showed that PE and GDM did not affect the expression of *SLC18A2* lncRNA and mRNA (Fig. 5a2, a3), indicating that these two pregnancy complications do not affect the function of the nervous system and the monoamine transport pathway. *SLC18A* monoamine *2* is a vesicular monoamine transporter that acts to accumulate cytosolic into SVs, using the proton gradient maintained across the synaptic vesicular membrane. Dysfunction in the activity of the monoaminergic system has been implicated in several human neuropsychiatric disorders [29] and is known to influence cognitive outcomes after traumatic a brain injury [30].

*SYT15* and *SYT9* encode the synaptotagmin family of membrane trafficking proteins, which are involved in the trafficking and exocytosis of secretory vesicles [31]. The *STX8* gene is involved in protein trafficking from early-to-late endosomes via vesicle fusion and exocytosis. The *STX8* gene encodes a vesicle trafficking protein that functions in the early secretory pathway, possibly by mediating retrograde transport from *cis*-Golgi membranes to the ER [32]. The *SV2C* gene encodes synaptic vesicle glycoprotein 2C, which regulates the secretion of neural and endocrine cells. The *SV2C* gene selectively enhances low-frequency neurotransmission and positively regulates vesicle fusion by maintaining the readily releasable pool of secretory vesicles [33]. The *SYP* gene encodes the synaptic protein, synaptophysin, an integral membrane protein of small SVs in the brain and endocrine cells. Synaptophysin is a calcium ion–binding protein that may also bind cholesterol and is thought to be involved in direct targeting of vesicle-associated membrane protein 2 (synaptobrevin) to intracellular compartments [34].

sPTB is a leading cause of newborn deaths and the second leading cause of deaths worldwide among children < 5 years of age [35]. Many studies have investigated the etiology of sPTB, but fewer studies have investigated the consequence of brain damage in children born preterm. In this study a method for detecting maternal lncRNAs and mRNAs in EXs was developed to determine the influence on neuronal function. We found a significant effect of *SLC18A2* and *STX8* in sPTB compared with controls. sPTB may affect the function of SVs and hence the transmission of synaptic signals, thus causing nervous system dysfunction via SLC18A2 and STX8. Hypertension during pregnancy is the major cause of maternal and fetal morbidity and death. The most severe form is PE, which occurs in 6–8% of all pregnancies [36]. In many epidemiologic studies, PE is associated with an increased onset of cardiovascular and metabolic diseases in the later lives of the offspring [37]. The presence of PE during fetal life can lead to an increased risk of chronic non-communicable diseases in later life [38]. The effect of PE on the development of synaptic transmission in the fetal nervous system is not known. Our results showed that pregnant women with PE have an abnormal expression of *STX8* lncRNA and mRNA, while the exact correlation between further examination. The rate of GDM has been increasing, but little is known about the long-term complications of GDM in offspring. Evidence from epigenetic studies strongly advocates for the need to identify neuropsychiatric complications among offspring who are exposed to GDM. Several researchers have suggested that there is a possible association between in utero exposure to GDM and ASD among offspring [39]. In GDM patients, the expression of *SV2C* lncRNA/mRNA and of *SYP* mRNA was higher than healthy pregnant women (Fig. 5d3, f3), indicating that GDM affects the release of fetal SVs through the SV2C and SYP pathways.

## Limitations

A challenge of this study was that the total EXs we isolated from the maternal circulation were mainly derived from trophoblasts. Although neuronal markers (LICAM and NES) have been shown to be present within the total EXs, the portion of neuronally-derived EXs in peripheral circulation is unknown. Obtaining neuronal EXs as derived from fetuses would be even more difficult. This has limited application of SVATs as the specific biomarker for prenatal screening and risk assessment for autism or other neurologic disorders during pregnancy.

## Conclusions

In summary, our results documented that exosomal lncRNAs are correlated with corresponding mRNA in healthy pregnant women. EXs can cross the blood–brain barrier and hence can serve as a marker of brain health. Thus, lncRNAs that are associated with neurologic development found in EXs can be detected in plasma. Indeed, this work demonstrated exosomal presynaptic-related lncRNAs and mRNAs in blood plasma of pregnant women. The early manifestations of abnormal molecular contents often have potential early warning effects. Examination of the expression patterns of VASTs in maternal EXs during pregnancy and tracking their changes may provide reference for understanding the early development of neural circuits in fetal brain and provide valuable information for assessment of the brain function after birth.

## Supplementary Information

**Additional file 1:** **Table S1**. Primers used for qRT-PCR.

## Data Availability

All data generated or analyzed during this study are included in this published article and its supplementary information files.

## Abbreviations

SV: Synaptic vesicle
SVATs: SV-associated transcripts
GEP: Gene expression profile
ASD: Autism spectrum disorder
GW: Gestational week
sPTB: Spontaneous preterm birth
PE: Preeclampsia
GDM: Gestational diabetes mellitus
EXs: Exosomes
SVAPs: Synaptic vesicle-associated proteins
SVC: Synaptic vesicle cycling
sPTL: Spontaneous preterm labor
pPROM: Preterm premature rupture of membrane
DEPs: Differential expression profiles
NTA: Nanoparticle tracking analysis
