# Supplementary material for: Autism-associated synaptic vesicle transcripts are differentially expressed in maternal plasma exosomes of physiopathologic pregnancies
Source: J Transl Med. 2021 Apr 15;19:154. doi: 10.1186/s12967-021-02821-6 (PMC8051067; doi:10.1186/s12967-021-02821-6)
Supplement: Supplementary file 1 — Additional file 1: Table S1. Primers used for qRT-PCR. [file 12967_2021_2821_MOESM1_ESM.docx]

| **Gene symbol** | **lncRNA** | **mRNA** |
| --- | --- | --- |
| ***SYT15*** | F(5’-3’):CCAGGAGCATCCAAGCAGAT | F(5’-3’):CATGTATGGTGGGGGCCATC |
|  | R(5’-3’):CCCCAGCTTGATCCAATCCC | R(5’-3’):ATTCCACCGAGAACCACAGC |
| ***STX8*** | F(5’-3’):GAGTCTGCAGGATGGCACC | F(5’-3’):CAGCTTGAAGGGGACCGAAG |
|  | R(5’-3’):ACTCATCAGGCTGGACCTTTG | R(5’-3’):TTCACTCATCAGGCTGGACC |
| ***SYT9*** | F(5’-3’):AATAGTGACCCCAGGCTTGC | F(5’-3’):CCCTGGTGGAGAAACGATGA |
|  | R(5’-3’):GCTTCTTCAGCTCGGTCCTT | R(5’-3’):AAGGCCAGTTAGTGCTGTGG |
| ***SYP*** | F(5’-3’):CAAGGCTGCCCATCAATGAC | F(5’-3’):AGTGCGCTAGAGCATTCTGG |
|  | R(5’-3’):CTTCAGACCTTCCCACCAGTC | R(5’-3’):TCTGCCTCGCTTAAAGCCTC |
| ***SLC18A2*** | F(5’-3’):TAGCAGAAGAGTGGGCTTGT | F(5’-3’):CGGGAATGCTACCAGAGACC |
|  | R(5’-3’):GTCTCTGCAAGGGAGTAGGG | R(5’-3’):TCTTCACTGGGACAGTCGGA |
| ***SV2C*** | F(5’-3’):GTCCCAATCCCTGGTGTTGA | F(5’-3’):CCCACAAAGTGAAGTAACCAAGT |
|  | R(5’-3’):GTGTCACTCCTAGCGCTTCA | R(5’-3’):CCACACCCACTAATGGCTCC |
| ***U6*** | F(5’-3’): GTGCTCGCTTCGGCAGCAC | N.A. |
|  | R(5’-3’):AAAATATGGAACGCTTCACGAATT |  |

**Table S1: Primers used for qRT-PCR**
